# PFKP Activation Ameliorates Foot Process Fusion in Podocytes in Diabetic Kidney Disease

**DOI:** 10.3389/fendo.2021.797025

**Published:** 2022-01-14

**Authors:** Zongwei Zhang, Wei Liang, Qiang Luo, Hongtu Hu, Keju Yang, Jijia Hu, Zhaowei Chen, Jili Zhu, Jun Feng, Zijing Zhu, Qingjia Chi, Guohua Ding

**Affiliations:** ^1^ Division of Nephrology, Renmin Hospital of Wuhan University, Wuhan, China; ^2^ Nephrology and Urology Research Institute of Wuhan University, Wuhan, China; ^3^ The First College of Clinical Medical Science, China Three Gorges University, Yichang, China; ^4^ Department of Mechanics and Engineering Structure, Wuhan University of Technology, Wuhan, China

**Keywords:** PFKP, diabetic kidney diseases, glycolysis, podocyte injury, cytoskeletal remodeling, FBP

## Abstract

**Background:**

Glycolysis dysfunction is an important pathogenesis of podocyte injury in diabetic kidney disease (DKD). Foot process fusion of podocytes and increased albuminuria are markers of early DKD. Moreover, cytoskeletal remodeling has been found to be involved in the foot process fusion of podocytes. However, the connections between cytoskeletal remodeling and alterations of glycolysis in podocytes in DKD have not been clarified.

**Methods:**

mRNA sequencing of glomeruli obtained from db/db and db/m mice with albuminuria was performed to analyze the expression profiling of genes in glucose metabolism. Expressions of phosphofructokinase platelet type (PFKP) in the glomeruli of DKD patients were detected. Clotrimazole (CTZ) was used to explore the renal effects of PFKP inhibition in diabetic mice. Using *Pfkp* siRNA or recombinant plasmid to manipulate PFKP expression, the effects of PFKP on high glucose (HG) induced podocyte damage were assessed *in vitro*. The levels of fructose-1,6-bisphosphate (FBP) were measured. Targeted metabolomics was performed to observe the alterations of the metabolites in glucose metabolism after HG stimulation. Furthermore, aldolase type b *(Aldob)* siRNA or recombinant plasmid were applied to evaluate the influence of FBP level alteration on podocytes. FBP was directly added to podocyte culture media. Db/db mice were treated with FBP to investigate its effects on their kidney.

**Results:**

mRNA sequencing showed that glycolysis enzyme genes were altered, characterized by upregulation of upstream genes (*Hk1, and Pfkp*) and down-regulation of downstream genes of glycolysis (*Pkm, and Ldha*). Moreover, the expression of PFKP was increased in glomeruli of DKD patients. The CTZ group presented more severe renal damage. *In vitro*, the *Pfkp* siRNA group and ALDOB overexpression group showed much more induced cytoskeletal remodeling in podocytes, while overexpression of PFKP and suppression of ALDOB *in vitro* rescued podocytes from cytoskeletal remodeling through regulation of FBP levels and inhibition of the RhoA/ROCK1 pathway. Furthermore, targeted metabolomics showed FBP level was significantly increased in HG group compared with the control group. Exogenous FBP addition reduced podocyte cytoskeletal remodeling and renal damage of db/db mice.

**Conclusions:**

These findings provide evidence that PFKP may be a potential target for podocyte injury in DN and provide a rationale for applying podocyte glycolysis enhancing agents in patients with DKD.

## Introduction

Diabetic kidney disease (DKD) is one of the most common and severe microvascular complications of diabetes. It is the leading risk factor for progression to end-stage renal disease (ESRD) in patients with renal failure. DKD is the primary cause of death in people with diabetes. A total of 3-40% of patients with type 1 diabetes (T1DM) or type 2 diabetes (T2DM) develop DKD, accounting for 30-47% of ESRD (1, 2). However, existing treatments for DKD are extremely limited once proteinuria occurs at the early stage (3). Thus, the identification of treatment targets for DKD is urgently needed.

The presence of microalbuminuria is marked by a urinary albumin creatinine ratio (ACR) greater than 30 mg/g, and it is a marker of early renal damage in diabetic patients (4). Podocytes have a close relationship with proteinuria because they are located outside the glomerular basement membrane (GBM) (5). Hyperglycemia is the most prominent and essential feature of diabetes, and it can lead to podocyte damage through various mechanisms, including affecting podocyte metabolism and cytoskeletal structures (6).

One study suggested that both anaerobic glycolysis and aerobic glycolysis are crucial energy resources for podocytes (7). Besides, previous studies indicated that both glycolysis processes were altered in DKD (8, 9). Notably, one study determined that pyruvate kinase muscle type 2 (PKM2) deletion in podocytes accelerated DKD progression (10), proving that glycolysis plays a critical role in podocytes.

Podocytes exhibit the unique cytoskeletal architecture that is fundamentally linked to their function in maintaining the kidney filtration barrier. In addition, cytoskeletal architecture can regulate the podocyte shape, adhesion, stability, structure, slit diaphragm insertion, plasticity, and dynamic response to environmental stimulations (5). Therefore, even slight impairment of the podocyte cytoskeletal apparatus can result in foot process fusion in podocytes and proteinuria, finally leading to glomerular disease, including DKD (6, 11). Hence, it is vital to maintain the normal structure and function of the podocyte cytoskeleton.

Many glycolytic enzymes anchor to the cytoskeleton, thus, enzymes and the cytoskeleton are closely related (12). Movement of the cytoskeleton can affect the activity of glycolytic enzymes (13), and glycolytic enzymes can also affect the podocyte cytoskeleton by regulating glucose metabolism (12). However, the interactions between glycolytic enzyme and podocyte cytoskeleton have not been reported. Here, we found that the glycolytic enzyme PFKP can protect the normal cytoskeletal structure of podocytes through its catalyzed metabolite fructose-1,6-bisphosphate (FBP).

## Materials and Methods

### Animal Studies

Db/db and db/m male mice (8-9 weeks, 20-40 g) were obtained (CAWENS, Changzhou, China) and housed in specific pathogen-free conditions with unrestricted access to food and water at the Center for Animal Experiments of Wuhan University. All protocols were approved by the Animal Ethics Review Board of Wuhan University and performed in accordance with the guidelines of the National Health and Medical Research Council of China (No.20200306). For the mRNA sequencing samples, db/db and db/m male mice (n=30 each) were raised from 8 weeks to 24 weeks, and kidney samples were harvested at 16 weeks and 24 weeks. The mice were randomly divided into four groups (n=6 each), db/m+0.1% DMSO (D2650, Sigma-Aldrich, USA), db/db+0.1% DMSO, db/m + Clotrimazole (CTZ) (HY-10882, MCE, China), and db/db + CTZ group. The dosage of CTZ is 100 mg/kg/day for two weeks by intraperitoneal injection in db/m + CTZ and db/db + CTZ group since 24 weeks (14). Besides, the mice were divided into another six groups randomly (n=6 each), db/m+ saline (Baxter, China), db/db+ saline, db/m + FBP (500 or 1000 mg/kg/day) (SC-214805, Santa cruz), and db/db + FBP (500 or 1000 mg/kg/day) group. The dosage of FBP is 500 or 1000 mg/kg/day for two weeks by intraperitoneal injection in db/m + FBP and db/db + FBP group beginning at 24 weeks (15, 16). 24-hour urine samples were collected in metabolic cages, and the urine ACR was measured every two weeks. The animals were sacrificed, and their kidneys were perfused with physiological saline before isolation and storage at -80°C for biochemical and renal pathological analysis.

### Glomerular Isolation, mRNA Sequencing and Data Analysis

Renal cortices from 24-week-old db/m and db/db mice were minced and digested with collagenase. Glomeruli were isolated by successive sieving through 100-, 70-, and 40- μm mesh sieves. The glomeruli-rich preparation retained on the 40- μm strainer was rinsed into a cell culture dish, and then glomeruli were collected into 15ml centrifuge tubes. After centrifugation, glomeruli were collected into cryopreservation tubes and stored at -80°C. Total RNA extraction from glomeruli was performed with Trizol reagent (Invitrogen, USA), according to the manufacturer’s instructions. The RNA concentration was calculated by a NanoDrop spectrophotometer. mRNA sequencing analysis was performed by Myhalic Biotechnoligical Co., Ltd. (Wuhan, China). RNA-seq raw data and processed data were uploaded to the GEO database (GSE184836). Differentially expressed genes were determined using DESeq2 (adjusted *P* < 0.05). Heatmaps of differentially expressed genes identified in our study were generated by TBtools (17).

### Targeted Metabolomics

The harvested podocytes were stored in the refrigerator at -80°C, and the samples were taken out. 1mL methanol acetonitrile aqueous solution (2:2:1, V/V) was added, followed by 60s vortex and low-temperature ultrasound for 30min, twice, at -20°C. The precipitated protein was placed for 1h, and 14000RCF was centrifuged at 4°C for 20min. The supernatant was freeze-dried, and the samples were stored at -80°C. The samples were separated by Agilent 1290 Infinity LC ULTRA performance liquid chromatography system. Mobile phase: Liquid A was 10 mM ammonium acetate aqueous solution, and liquid B was acetonitrile. The samples were placed in an automatic sampler at 4°C, the column temperature was 45°C, the flow rate was 300 μL/min, and the injection volume was 2 μL. A 5500 QTRAP mass spectrometer (AB SCIEX) was used for mass spectrometry analysis in negative ion mode. 5500 QTRAP ESI source conditions are as follows: Source Temperature 450°C, Ion Source Gas1(Gas1):45, Ion Source Gas2(Gas2):45, Curtain Gas (CUR):30, ionSapary Voltage Floating(ISVF)-4500 V; MRM mode was adopted to detect the ion pairs to be measured, and the ion pairs information of all energy metabolites was shown in **Supplementary Data Set 1**. The peak area and retention time were extracted by Multiquant software. The energy metabolite standard was used to correct the retention time and identify metabolites.

### Human Renal Samples

Renal biopsy samples from patients diagnosed with DKD according to the ADA and KDOQI guideline (18, 19) (3 males and 3 females; age, 45–55 years; mean age, 49.17 ± 3.43 years) were obtained from the Division of Nephrology, Renmin Hospital of Wuhan University, Wuhan, China. The control samples (3 from males and 3 from females; age, 37–56 years; mean age, 46.17 ± 6.49 years) were para-carcinoma tissues from individuals without other renal diseases who had tumor nephrectomies, and were obtained from the Division of Pathology, Renmin Hospital of Wuhan University, Wuhan, China. The detail clinical information of these patients was shown in **Table S1**. This investigation was performed in accordance with the principles of the Declaration of Helsinki. The experiment was performed in accordance with the approved guidelines of Wuhan University and was approved by the Research Ethics Committee of Renmin Hospital of Wuhan University. Informed consent was received from the patients.

### Reagents and Antibodies

NaCl solution (0.9%) was purchased from Baxter China. Anti-PFKP antibody (GTX35238, 1:100-1:1000), PKM2 (CST, 4053, 1:1,000), HK1(CST, 2024,1:800) and LDHA(Sigma Aldrich, QC52376, 1:1000) were used for Western blotting, immunofluorescence (IF) staining and immunohistochemical (IHC) staining. The primary antibodies for Western blotting against PFKM (ab154804, 1:1000), and PFKL (ab45688, 1:1000) were all from Abcam (Cambridge, MA). The primary antibodies for Western blotting against ROCK1 (21850-1-AP, 1:1000), RhoA (10749-1-AP, 1:1000), α-tubulin (11224-1-AP, 1:1000), ALDOB (18065-1-AP,1:1000), and alpha-tubulin(11224-1-AP,1:1000) were purchased from proteintech (Wuhan, China). Anti-GAPDH (sc-365062, 1:1000) was purchased from Santa Cruz (Santa Cruz, CA). DAPI and secondary antibodies against rabbit IgG-HRP(ANT020), IgG-HRP (ANT019), Alexa Fluor 488, 594 conjugated anti-mouse IgG, and anti-rabbit IgG were obtained from Antgene (Wuhan, China).

### Histological and Immunohistochemistry Examination

Kidney tissues were systemically perfused with cold PBS and 4% paraformaldehyde (pH 7.4). Harvested kidneys were processed by a standard protocol for histological examination. Briefly, the tissues were embedded in paraffin, sectioned at 5 µm, stained with periodic acid-Schiff stain (PAS), and observed and photographed under a microscope (Olympus, Tokyo, Japan). For IHC staining, sections were incubated with the indicated primary antibodies. Five visual fields from individual groups were randomly selected to determine the percentage of positive staining area using ImageJ analysis software.

### Cell Culture and Transfection

Conditionally immortalized human podocytes kindly provided by Dr. Moin A. Saleem (Academic Renal Unit, Southmead Hospital, Bristol, UK) were cultured under standard conditions. The medium consisted of RPMI 1640 (HyClone, USA) containing 10% heat-inactivated fetal bovine serum (FBS; Gibco, USA), 100 U/mL penicillin G, 100 μg/mL streptomycin (Invitrogen, USA) and 1× insulin-transferrin-selenium (ITS; Invitrogen, USA) at 33°C. To induce differentiation, podocytes were cultured at 37°C for 10-14 days without ITS, and differentiated podocytes were used in all experiments. Differentiated podocytes were stimulated with 30 mM high glucose (HG) for 24 h. For siRNA transfection, we performed HiPerFect transfection (Qiagen, Germany) according to the manufacturer’s protocol with *Pfkp*, and *Aldob* siRNA and control siRNA (Qiagen). Briefly, cells were liberated, seeded into 6-well plates and transfected with serum-free medium containing 100 nM Pfkp siRNA or control siRNA for 6-8 hours until reaching 70% confluence, after which the cells were recovered in complete medium and 30 mM HG when necessary. The sequence of human *Pfkp* siRNA oligonucleotides in this study was 5′-GCAACGTAGCTGTCATCAA-3′. The sequence of human *Aldob* siRNA oligonucleotides in this study was 5′-CCAGAGCATTGTTGCCAAT-3′. To overexpress PFKP, transfection of the *Pfkp* plasmid (GeneChem, Wuhan, China) was conducted with X-tremeGENE HP DNA Transfection Reagent (Roche) according to the manufacturer’s instructions. A density of 2×10^5^ cells was first seeded into each well of a 6-well plate and then transfected with complexes containing 2 μg of Pfkp plasmid or a negative control with pcDNA3.1 and 2 μl of the X-tremeGENE transfection reagent. Then, the cells were incubated under normal conditions for 24 h at 37°C, recovered in complete medium and stimulated with 30 mM glucose (HG) as necessary. Similarly, the *Aldob* plasmid (GeneChem, Wuhan, China) was used to overexpress ALDOB. FBP (SC-214805, Santa Cruz) was added to complete medium at 5 mM or 10 mM. Each experimental result was confirmed in three independent podocyte clones.

### ATP Production

After treatment, the intracellular ATP content was assessed using a luciferase ATP detection assay kit (Beyotime, China) according to the manufacturer’s protocol. Briefly, the cells were harvested and lysed. Then, the protein content of the supernatant was determined with a BCA protein kit (Beyotime, China), and the protein supernatant was mixed with ATP detection solution. Finally, the luminescence of each sample was measured by a fluorescence microplate reader.

### PFK Enzymatic Activity

The enzymatic activity of PFK was determined using a kit (BC0530, Solarbio, China) according to the manufacturer’s protocol. Since PFK catalyzes the conversion of fructose-6-phosphate and ATP to FBP and ADP, and Pyruvate kinase and lactate dehydrogenase further catalyze the oxidation of NADH to NAD^+^, the decrease rate of NADH was measured at 340 nm to detect PFK activity and the absorbance of each sample was analyzed by a fluorescence microplate reader.

### FBP Level

The level of FBP was measured using a kit (BC2240, Solarbio, China) according to the manufacturer’s protocol. The reaction of fructose 1,6-diphosphate with 2,4-dinitrophenylhydrazine catalyzed by aldolase in acidic medium produces 2,4-dinitrophenylhydrazone, which is reddish brown in alkaline solution, has a characteristic absorption peak at 540 nm, and could indicate the level of FBP. A fluorescence microplate reader analyzed the absorbance of each sample.

### Wound-Healing Assay

Podocytes were seeded into 12-well plates to reach 80% confluence. Then, a wound was created by scratching with a pipette tip, and the cultures were washed with PBS to remove cell debris. Cells stimulated with HG were visualized by light microscopy at 0 h, 24 h, and 48 h (3 separate fields/well). Covered surface areas were measured by ImageJ.

### Cytoskeleton Staining

Phalloidin (ab176753, Abcam, Cambridge, MA) stained the cell cytoskeleton. After treatment as described above, the cells were washed with PBS, fixed in 4% paraformaldehyde, and blocked with 5% bovine serum albumin (BSA). Then, the cells were stained with phalloidin for 60 min. After three washes, the nuclei were stained with DAPI. Finally, the results were observed *via* fluorescence microscopy. A normal podocyte cytoskeleton structure contains F-actin distributed as obvious homogenous bundles that traverse the cell along the axis of the podocyte. Cytoskeletal remodeling signs include F-actin assembly in cortical regions, agminated F-actin along the podocyte periphery, and a slight diffuse F-actin cytoplasmic distribution (20).

### Western Blotting

After treatment, cells or kidney tissue were homogenized in RIPA lysis buffer with PMSF and protease inhibitor cocktail (Roche) for 30 min at 4°C. Total proteins were separated in an 8-10% SDS-PAGE gel and transferred onto PVDF membranes. Then, the membranes were blocked with 5% milk for 1h. After blocking with milk, the membranes were incubated with primary antibodies (PFKP, PFKM, PFKL, PKM2, ALDOB, ROCK1, RhoA, α-tubulin and GAPDH) overnight at 4°C. The next day, the membranes were incubated with a secondary antibody (Antgene, China). After washing the membranes three times, bands were revealed by an ECL chemiluminescent kit (Biosharp, China). Finally, the bands were analyzed using a ChemiDocTM MP Imaging system (Bio-Rad, USA).

### Immunofluorescence Staining

After the indicated treatment, cells and tissues were fixed with 4% paraformaldehyde and blocked with 5% BSA. Specific primary antibodies (PFKP) were then applied overnight at 4°C. Next, the samples were incubated with fluorescent secondary antibodies for 1 h. After washing, the nuclei of the samples were counterstained with DAPI. Fluorescence results were analyzed using a confocal laser microscope (Olympus, Japan).

### Real-Time PCR

Total RNA from cells and kidney tissue was prepared using TRIzol reagent (Invitrogen, USA). The concentration was measured by a NanoDrop spectrophotometer. Then, 1 µg RNA was retrotranscribed to cDNA using the PrimeScript RT Reagent Kit. PCR amplification was performed using a SYBR Green Kit (Takara, Japan) with the following conditions: 95°C for 30 s; followed by 40 cycles of application at 95°C for 5 s and 60°C for 30 s; and annealing at 60°C for 34 s. All primer sequences are shown in **Table S2**. The relative gene expression was quantified by the 2^−ΔΔCT^ method, and GAPDH was used as an endogenous control.

### Statistical Analyses

All experiments were repeated at least 3 times. Quantitative data were presented as the mean ± SD, and statistical analyses were performed using SPSS v22.0. Statistical comparisons of groups were performed using one-way ANOVA, and the least-significant difference (LSD) test was used for multiple comparisons. Differences for which *P*<0.05 were considered statistically significant.

## Results

### Renal Phenotype Alteration of db/db Mice and Profiling of Genes Involved in Glucose Catabolism in Glomeruli From db/db and db/m Mice by mRNA Sequencing

**Figure 1:**
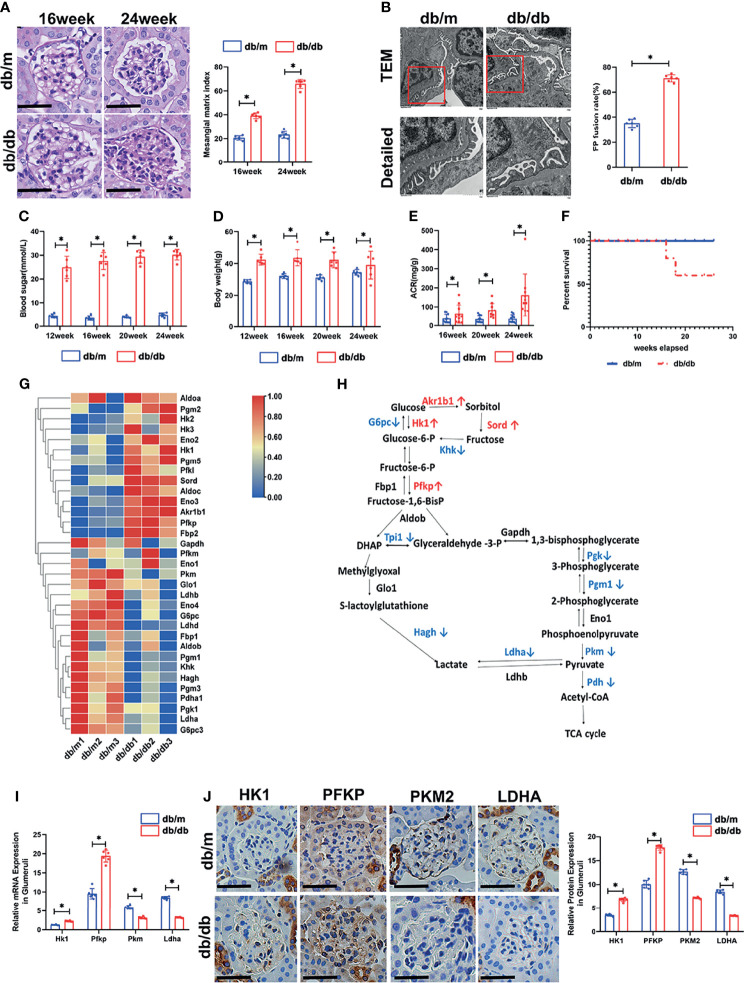
Renal phenotype alteration of db/db mice and profiling of genes involved in glucose catabolism in glomeruli from db/db and db/m mice by mRNA sequencing. **(A)** Representative microscopy images and quantification of PAS staining of kidney sections from db/m and db/db mice at 16 and 24 weeks of age (original magnification ×400). **(B)** Representative findings of the ultrastructure of capillary loops collected from db/m and db/db mice at different timepoints, as examined by transmission electron microscopy (original magnification×8,000, ×12,000). **(C)** Blood sugar of db/m and db/db mice. **(D)** Body weight of db/m and db/db mice. **(E)** Quantitative analysis of ACR (albumin-to-creatinine ratio) in db/m and db/db mice. **(F)** Survival curve of db/m and db/db mice over time. **(G)** Gene expression profiles were compared between glomeruli of db/m and db/db mice, and heat maps were generated based on significantly differential expression of genes related to glucose metabolism. **(H)** Schematic flow illustrating the representative genes in the glycolysis cascade. Red text represents upregulated genes, and blue text represents downregulated genes in the glomeruli of db/db mice in comparison to db/m mice. Akr1b1, aldo-keto reductase family 1, member B1 (aldose reductase); Hk, hexokinase; G6pc, glucose-6-phosphatase, catalytic subunit; Sord, sorbitol dehydrogenase; Pfk, phosphofructokinase; Fbp2, fructose-1,6-diphosphatase 2; Gapdh, glyceraldehyde 3-phosphate dehydrogenase; Tpi1, triosephosphate isomerase 1; Pgk, phosphoglycerate kinase; Pgm1, phosphoglucomutase-1; Eno1, enolase 1; Pkm, pyruvate kinase isoenzyme; Pdh, pyruvate dehydrogenase; Ldh, lactate dehydrogenase; Hagh, hydroxyacyl glutathione hydrolase; Glo1, glyoxalase 1. **(I)** Relative mRNA expression of Hk1, Pfkp, Pkm and Ldha in the glomeruli of db/m and db/db mice determined by real-time PCR. **(J)** Representative immunohistochemistry staining of glomerular HK1, PFKP, PKM2, and LDHA in db/m and db/db mice. For all figures, n=6 independent experiments/group. In all statistical plots, *P < 0.05. Scale bars: 20 µm, HK1, hexokinase 1; PFKP, phosphofructokinase 1 platelet type; PKM2, pyruvate kinase isoenzyme 2; LDHA, lactate dehydrogenase A.

To profile the altered genes in db/db mice compared with db/m mice, the renal phenotype changes of db/db mice were observed by PAS staining (**Figure 1A**) and transmission electron microscopy (**Figure 1B**), blood sugar test results (**Figure 1C**), body weights (**Figure 1D**), and ACR values (**Figure 1E**) were systemically evaluated. PAS staining indicated dilated mesangial matrix in the glomeruli of db/db mice. Electron microscopy showed that podocyte foot processes were fused, and the glomerular basement membrane was thickened in db/db mice compared with db/m mice. Additionally, db/db mice presented higher blood sugar levels, body weights, ACR values, and mortality rates (**Figure 1F**). Subsequently, the isolated glomeruli were subjected to mRNA sequencing. Glucose metabolism-related genes were systemically evaluated between db/db mice and db/m mice. Intriguingly, several upstream glycolysis genes were upregulated, including *Pfkp* and *Hk1*. Conversely, downstream glycolysis genes were downregulated, including *Pkm*, *Ldha*, *Pdh*, etc. (**Figures 1G, H**). To further confirm the pattern of glycolysis in DKD, the mRNA expression of *Hk1*, *Pfkp*, *Pkm*, and *Ldha* in mouse glomeruli was evaluated by qPCR. Consistent with the sequencing results, *Hk1* and *Pfkp* gene expression was elevated in the glomeruli of db/db mice compared with db/m mice, while *Pkm* and *Ldha* expression was decreased, as shown in **Figure 1I**. Additionally, the protein expression pattern was confirmed by immunohistochemical staining in the glomerular area (**Figure 1J**).

### PFK Expressions in db/m and db/db Mice

**Figure 2:**
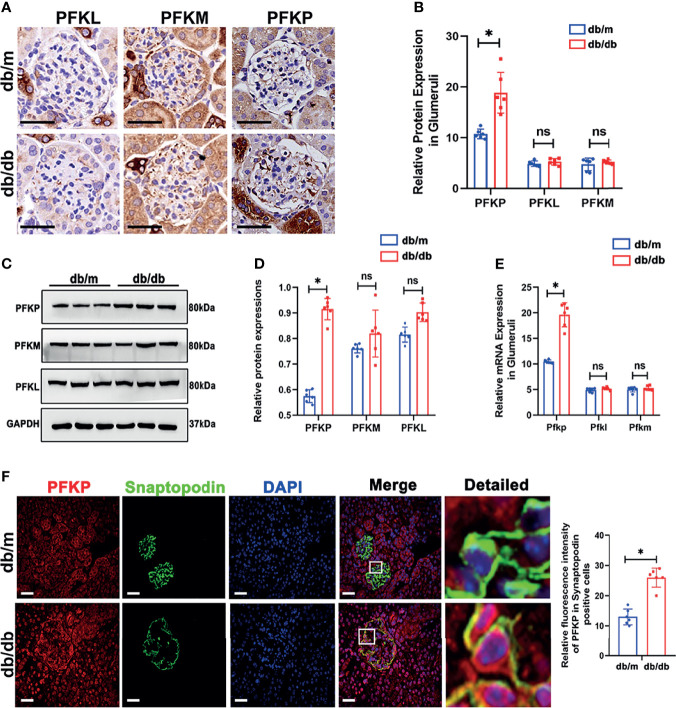
PFK expressions in db/m and db/db mice. **(A, B)** Representative immunohistochemistry staining of glomerular PFKP, PFKM and PFKL in db/m and db/db mice. **(C, D)** Western blotting analysis of the expression of PFKP, PFKM and PFKL in glomeruli of db/m and db/db mice. GAPDH was used as the loading control. **(E)** Relative mRNA expression of *Pfkp, Pfkm* and *Pfkl* in glomeruli of db/m and db/db mice determined by real-time PCR. **(F)** Representative immunofluorescent staining of PFKP in podocyte of glomeruli from db/m and db/db mice. Synaptopodin was used as podocyte markers. For all figures, n=6 independent experiments/group. In all statistical plots, *P < 0.05, ns, No significance. Scale bars: 20 µm, PFKP, phosphofructokinase 1 platelet type; PFKM, phosphofructokinase 1 muscle type; PFKL, phosphofructokinase 1 liver type.

The sequencing data suggests that the compromise of downstream glycolysis could promote the accumulation of intermediates in upstream glycolysis. As shown in **Figure 1H**, *Pfkp* connects the upstream and downstream portions of the glycolysis cascade. Hence, exploring *Pfkp* alteration in db/db mice was of importance. Since three subtypes of PFK1 have been reported in rodents, all three subtypes were evaluated in the glomeruli. Interestingly, PFKP showed elevated expressions in db/db glomeruli, while the expression of PFKL and PFKM remained comparable according to IHC staining (**Figures 2A, B**). The protein (**Figures 2C, D**) and mRNA (**Figure 2E**) levels of PFKP in the glomeruli of db/db mice and db/m mice were verified by western blotting and q-PCR, respectively. Double staining of glomeruli indicated that PFKP was highly enriched in podocytes from db/db mice (**Figure 2F**).

### PFKP Expression in Podocytes From Patients With Biopsy-Proved DKD

**Figure 3:**
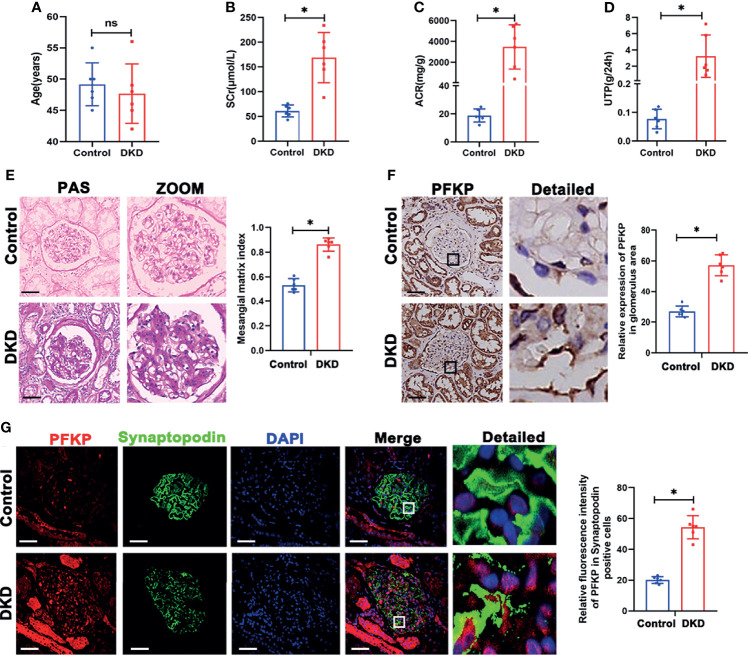
PFKP expression in podocytes from patients with biopsy proved DKD. **(A)** Age of subjects in each group. **(B)** SCr values of subjects in each group. **(C)** ACR values of subjects in each group. **(D)** UTP values of subjects in each group. **(E)** Representative microscopy images and quantification of PAS staining of kidney sections in each group (original magnification ×200). **(F)** Representative immunohistochemistry staining of glomerular PFKP in each group. (Original magnification ×200). **(G)** Representative immunofluorescence staining of PFKP in podocytes in each group. Synaptopodin was used as podocyte markers. For all figures, n=6 independent experiments/group. In all statistical plots, *P < 0.05, ns, No significance. Scale bars: 20 µm, DKD, diabetic kidney disease; PFKP, Phosphofructokinase 1 platelet type; SCr, Serum creatinine; UTP, Total proteinuria; ACR, urine albumin/creatinine ratio.

Based on the above results, PFKP was activated in db/db mice. The expression profile of PFKP was tested in patients with biopsy-proved DKD. Clinical characteristics of DKD patients including age, serum creatinine (SCr), ACR, and total proteinuria (UTP) were presented in **Figures 3A**–**D**. Then, PAS stain showed the mesangial expansion and segmental glomerulosclerosis in glomeruli in patients with DKD (**Figure 3E**). PFKP staining in podocyte was enhanced in glomeruli from patients with DKD compared with that in control subjects. The above pattern was tested by IHC (**Figure 3F**) and double immunofluorescent staining approaches (**Figure 3G**) respectively.

### Suppression of PFKP Overactivation Aggravated Renal Damage in Diabetic Mice

**Figure 4:**
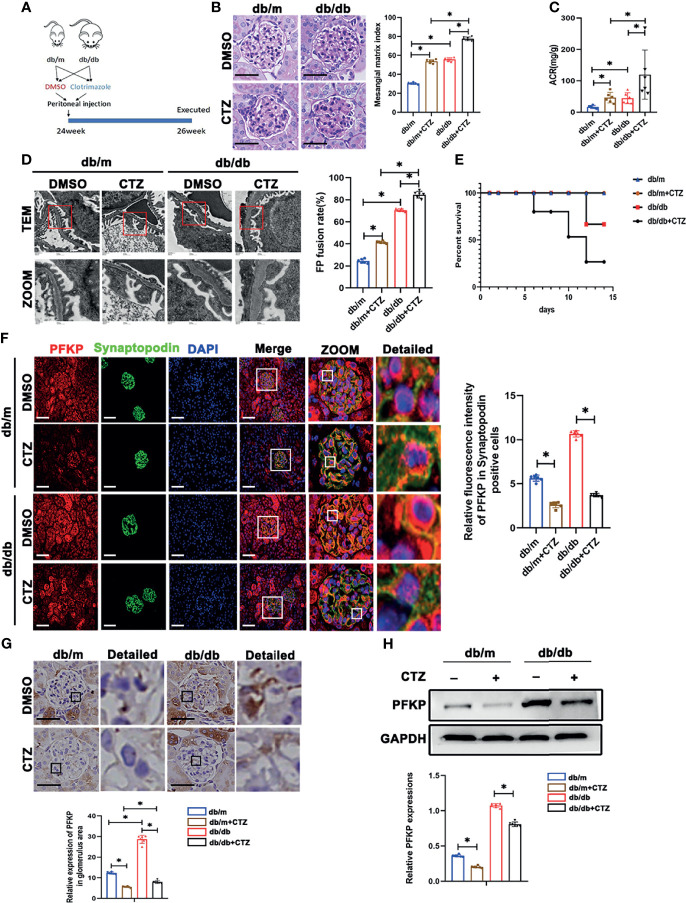
Suppression of PFKP overactivation aggravated renal damage in db/db mice. **(A)** Schematic diagram of Clotrimazole administration in advanced db/db mice. **(B)** Representative microscopy images and quantification of PAS staining of kidney sections in each group (original magnification ×400). **(C)** Quantitative of ACR (albumin-to-creatinine ratio) in each group. **(D)** Representative transmission electron microscopy images of the ultrastructure of capillary loops in each group (original magnification×8,000, ×12,000). **(E)** Survival curve of db/m and db/db mice treated with CTZ over time. **(F)** Representative immunofluorescence staining of podocytes PFKP in each group. Synaptopodin was used as the podocyte marker. **(G)** Representative immunohistochemistry staining of glomerular PFKP in each group. **(H)** Western blotting analysis of PFKP expression in glomeruli of each group. GAPDH was set as loading control. For all figures, n=6 independent experiments/group. *P < 0.05. Scale bars: 20 µm, CTZ, Clotrimazole; PFKP, Phosphofructokinase 1 platelet type.

To further investigate the function of PFKP in podocytes, db/db and db/m mice at 24 weeks of age were administrated intraperitoneally with CTZ for two weeks to inhibit the activity of PFKP (21) (**Figure 4A**). The same volume of solvent with 0.1% DMSO was injected as the control group. No significant changes in body weight and blood sugar were found in CTZ-administrated mice compared with solvent-treated animals. (**Figures S1**, **S2**). However, ACR was significantly higher in CTZ–administrated db/db mice when compared with solvent-treated db/db mice (**Figure 4C**). The pathologic glomerular sclerosis (**Figure 4B**) and foot process fusion by TEM (**Figure 4D**) were worsened in CTZ–administrated db/db mice compared with vehicle-treated db/db mice. Moreover, the mortality of db/db mice with proteinuria at 24-26week age was deteriorated by CTZ administration (**Figure 4E**). To a certain degree, CTZ administration promoted mild proteinuria (**Figure 4C**) and modest pathologic changes, including glomerular sclerosis (**Figure 4B**) and foot process fusion (**Figure 4D**) in db/m mice at comparable age with proteinuria-onset db/db mice. Conversely, the mortality rate was not impacted by CTZ administration in db/m mice. In order to identify the inhibition ability of CTZ on PFKP, IHC and immunofluorescence double staining were used to evaluate the expression of PFKP in glomeruli (**Figure 4G**) and podocyte (**Figure 4F**) respectively. In addition, western blotting analyses indicated that the innate and enhanced protein levels of PFKP were suppressed by CTZ administration in glomeruli from both db/m and db/db mice respectively (**Figure 4H**). Overall, CTZ accelerated podocyte damage under diabetic conditions, which indicates that PFKP may play a protective role in podocytes in DKD.

### High Glucose Promoted PFKP Expression and Cytoskeletal Remodeling in Podocytes *In Vitro*

**Figure 5:**
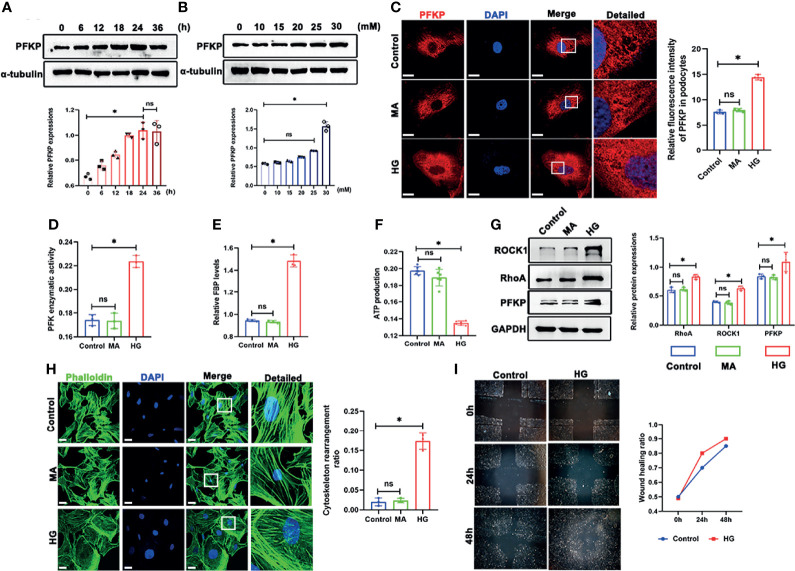
High glucose promoted PFKP expression and cytoskeletal remodeling in podocytes *in vitro*. **(A)** Representative western blots of PFKP expression in 30 mM HG-treated podocytes at various times points and quantification of these results, with α-tubulin as the loading control. **(B)** Representative western blots of PFKP expression in cultured podocytes stimulated with different concentrations of glucose for 24 h and quantification of these results, with α-tubulin set as the loading control. **(C)** Representative immunofluorescence staining of podocytes PFKP in each group. **(D)** PFK enzymatic activity in each group. **(E)** FBP level in each group. **(F)** ATP production in each group. **(G)** Western blotting analyses of the expression of ROCK1, PFKP and RhoA in podocytes in each group. GAPDH was set as the loading control. **(H)** Cytoskeletal structures of podocytes labeled with phalloidin. **(I)** Motility of podocytes quantified by a wound-healing assay using an inverted microscope. For all figures, n=3 independent experiments/group. *P < 0.05, ns, No significance. Scale bars: 10 µm. HG, high glucose; MA, mannitol; ROCK1, Rho-associated, coiled-coil containing protein kinase 1; PFKP, phosphofructokinase 1 platelet type; RhoA, RhoA-GTPase.

The above CTZ administration indicated that PFKP potentially plays a protective role in podocytes in physical and stress conditions *in vivo*. However, the mechanisms remain to be elucidated. In subsequent experiments, the expression of PFKP was analyzed in podocytes exposed to HG *in vitro*. The three subtypes of PFK were evaluated in culturing podocytes *in vitro* (**Figures S3**, **S4**), and it was found that PFKP was the dominant subtype in podocytes in consist with *in vivo* studies. The expression of PFKP was markedly increased in a dose-dependent manner in the cultured podocytes (0, 10, 15, 20, 25 and 30mM HG) (**Figure 5B**) over 24h exposure. Similarly, a time-dependent increase of the PFKP protein level was exhibited in podocytes under HG (30mM) conditions (0, 6, 12, 18, and 24 h) (**Figure 5A**). In addition, HG-treated podocytes exhibited a dramatic increase in PFKP in the cytoplasm, as shown by immunofluorescence assays (**Figure 5C**). Furthermore, mannitol (MA) hypertonic control had no impact on PFKP expression and distribution in podocytes compared with normal glucose conditions (**Figure 5C**). In addition, PFK enzymatic activity and FBP levels were increased in HG-exposed podocytes (**Figures 5D, E**), while ATP production was decreased under HG exposure (**Figure 5F**). As PFKP was reported as a cytoskeleton binding protein, the cytoskeleton assembling pattern was evaluated in HG-exposed podocytes. Phalloidin labeling technique was performed to assess F-actin assembling in podocytes. Interestingly, F-actin rearrangement occurred over HG exposure in cultured podocytes. F-actin assembling model switched from being uniformly parallel with long axis of cell body in control group to being aggregating along cell edge in HG-exposed podocyte (**Figure 5H**). Moreover, the wound healing test indicated that HG exposure promoted podocyte migration than normal glucose (**Figure 5I**). RhoA/ROCK1 pathway was reported to be crucial in regulating cytoskeleton in podocytes (22). In line with F-actin remodeling over HG exposure, it was found that RhoA/ROCK1 pathway was activated in HG-exposed podocytes (**Figure 5G**).

### Deletion of PFKP Aggravated HG-Induced Cytoskeletal Remodeling in Podocytes *In Vitro*

**Figure 6:**
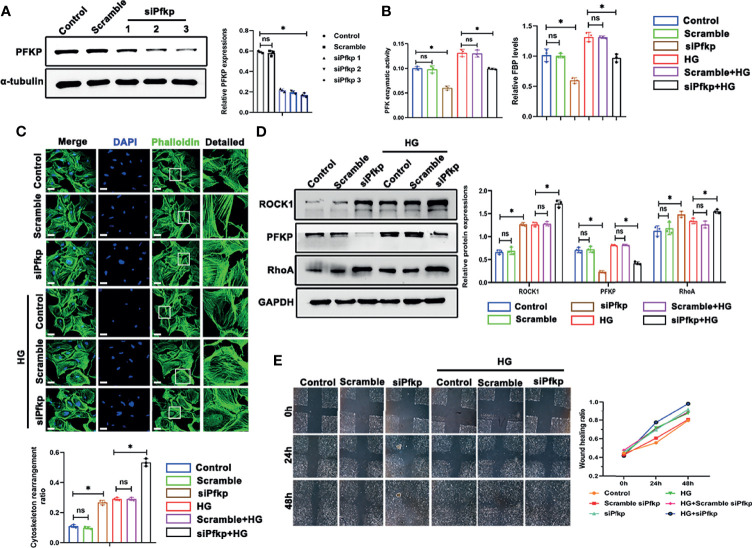
Deletion of PFKP aggravated HG-induced cytoskeletal remodeling in podocytes *in vitro*. **(A)** Western blots of PFKP expression after transfection with various *siPfkps*, with GAPDH set as the loading control. **(B)** PFK enzymatic activity and FBP level in each group. **(C)** Cytoskeletal structures of podocytes labeled with phalloidin and the cytoskeletal rearrangement ratio in each group (original magnification ×600). **(D)** Western blotting analyses of the expression of ROCK1, PFKP, and RhoA in podocytes of each group. GAPDH was set as the loading control. **(E)** Motility of podocytes measured by a wound-healing assay using an inverted microscope. For all figures, n=3 independent experiments/group. *P < 0.05, ns, No significance. Scale bars: 10 µm. HG, high glucose; ROCK1, Rho-associated, coiled-coil containing protein kinase 1; PFKP, phosphofructokinase 1 platelet type; RhoA, RhoA-GTPase.

To further investigate the impact of PFKP suppression on cytoskeletal assembling *in vitro*, siRNA interference was performed to silence *Pfkp* gene, and the efficiency of si*Pfkp* interference was confirmed by western blot analysis (**Figure 6A**). Accordingly, PFK enzymatic activity was suppressed and FBP level, the converted product of PFKP, was lower in *Pfkp-*downregulated podocytes (**Figure 6B**). In addition, suppressing PFKP by siRNA activated RhoA/ROCK1 signaling, magnified by HG exposure (**Figure 6D**). Morphologically, F-actin assemble remodeling was induced by suppression of PFKP in podocyte which was worsened by HG exposure (**Figure 6C**). Moreover, the migration ability of podocytes was enhanced by *Pfkp* interference and accelerated under HG conditions (**Figure 6E**).

### Overexpression of PFKP Ameliorated HG-Induced Cytoskeletal Remodeling in Podocytes *In Vitro*

**Figure 7:**
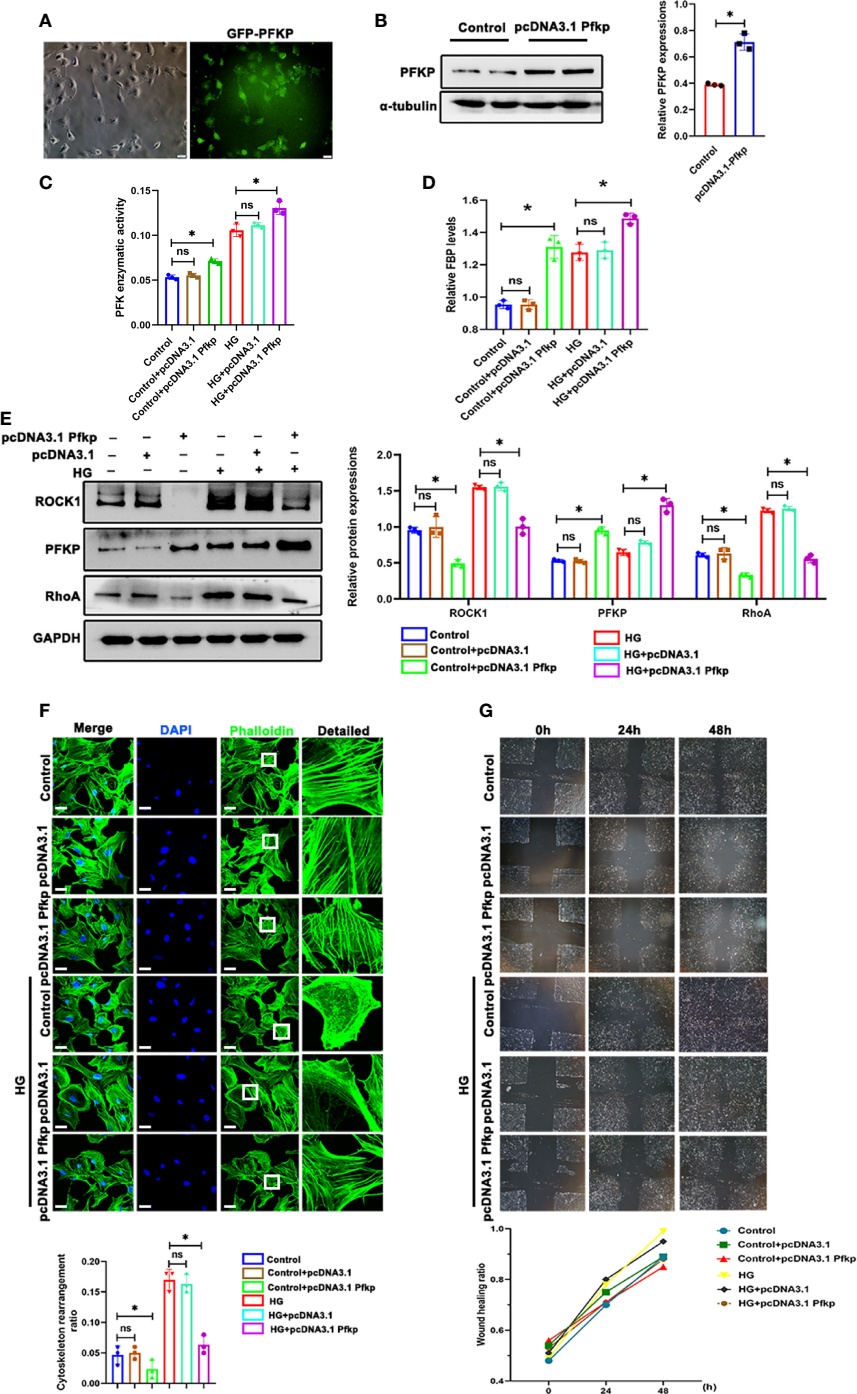
Overexpression of PFKP ameliorated HG-induced cytoskeletal remodeling in podocytes *in vitro*. **(A)** After recombinant plasmid (pcDNA3.1-Pfkp) transfection into podocytes, the expression of EGFP was observed by fluorescence microscopy. **(B)** Western blots of PFKP expression after transfection with pcDNA3.1-Pfkp, with GAPDH set as the loading control. **(C)** PFK enzymatic activity in each group. **(D)** FBP level in each group. **(E)** Western blotting analyses of the expression of ROCK1, PFKP, and RhoA in podocytes in each group. GAPDH was set as the loading control. **(F)** Cytoskeletal structures of podocytes labeled with phalloidin. **(G)** Motility of podocytes measured by a wound-healing assay using an inverted microscope. For all figures, n=3 independent experiments/group. *P < 0.05, ns, No significance. Scale bars: 10 µm. HG, high glucose; ROCK1, Rho-associated, coiled-coil containing protein kinase 1; PFKP, phosphofructokinase 1 platelet type; RhoA, RhoA-GTPase.

As described above, PFKP deletion aggravated podocyte migration under HG conditions. To further confirm the effect of PFKP on podocyte migration, a recombinant plasmid (pcDNA3.1-*Pfkp*) was transfected into podocytes to overexpress PFKP *in vitro.* The efficiency of plasmid transfection was confirmed by western blot analysis (**Figures 7A, B**). Accordingly, PFK enzymatic activity and FBP levels were increased in *Pfkp-*overexpressed cells (**Figures 7C, D**). Moreover, overexpression of PFKP prevented activation of RhoA/ROCK1 signaling and remodeling of F-actin assemble even in the presence of HG exposures (**Figures 7E, F**). In addition, the wound healing test showed that with pcDNA3.1-*Pfkp* transfection, podocyte migration and movement slowed (**Figure 7G**).

### Exogenous FBP Ameliorated HG-Induced Cytoskeletal Remodeling in Podocytes *In Vitro*

**Figure 8:**
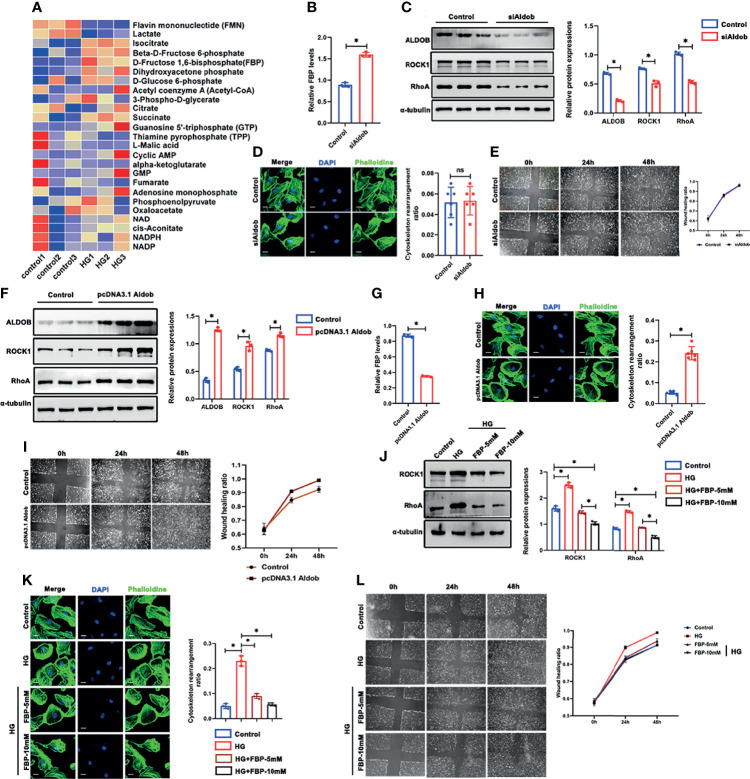
FBP ameliorated HG-induced cytoskeletal remodeling in podocytes *in vitro*. **(A)** Targeted metabolomics was performed between control and HG group of cultured podocytes, and heat maps were generated based on levels of the metabolites related to glucose metabolism. **(B)** Western blots of ALDOB, ROCK1, PFKP, and RhoA after transfected with siAldob, GAPDH was set as the loading control. **(C)** FBP level in control and siAldob group. **(D)** Cytoskeletal structure of podocytes labeled with phalloidin in control and siAldob group. **(E)** Motility ability of podocytes were measured by wound-healing assay using an inverted microscope in control and siAldob group. **(F)** Western blotting analyses the expression of ALDOB, ROCK1, PFKP, and RhoA in podocytes after transfected with pcDNA3.1-Aldob. GAPDH was set as loading control. **(G)** FBP level in control and pcDNA3.1-Aldob group. **(H)** Cytoskeleton structure of podocytes labeled with phalloidin in control and pcDNA3.1-Aldob group. **(I)** Motility ability of podocytes were measured by wound-healing assay using an inverted microscope in control and pcDNA3.1-Aldob group. **(J)** Western blotting analyses the expression of ALDOB, ROCK1, PFKP, and RhoA in podocytes after FBP treatment. GAPDH was set as loading control. **(K)** Cytoskeleton structure of podocytes labeled with phalloidin in each group. **(L)** Motility ability of podocytes were measured by wound-healing assay using an inverted microscope in each group. For all figures, n=3 independent experiments/group. *P < 0.05, ns, No significance. Scale bars: 10 µm. HG, high glucose; FBP, fructose-1,6-bisphosphate; ROCK1, Rho-associated, coiled-coil containing protein kinase 1; PFKP, Phosphofructokinase 1 platelet type; RhoA, RhoA-GTPase.

PFKP is the glycolytic enzyme that catalyzes the phosphorylation of fructose-6-phosphate (Fructose-6-P) to FBP(Fructose-1,6-P). The dynamic changes in PFKP and FBP levels suggest that PFKP may participate in cytoskeletal regulation by regulating FBP levels. To confirm that, targeted metabolomics was performed to observe the alterations of the metabolites of glucose metabolism. It showed that the level of isocitrate, fructose 6-phosphate, FBP, etc. were significantly increased, while the level of flavin mononucleotide and lactate decreased (**Figure 8A**). To further investigate the role of FBP in podocytes, we used aldolase (ALDO), which is the enzyme that catalyzes the breakdown of FBP, to control FBP levels in podocytes. First, the relative mRNA expression of the three subtypes of *Aldo* (*Aldoa*, *Aldob*, and *Aldoc*) in cultured podocytes was assessed by real-time PCR (**Figure S5**), which showed that *Aldob* was the dominant subtype in podocytes. With si*Aldob*, the RhoA/ROCK1 pathway declined as the level of FBP increased (**Figures 8B, C**). No significant cytoskeletal remodeling occurred in the si*Aldob* group (**Figure 8D**), and the motility of the podocytes was unaffected either (**Figure 8E**). In contrast, when transfected with a recombinant *Aldob* plasmid (pcDNA3.1-*Aldob*), FBP levels were decreased (**Figure 8G**), and the RhoA/ROCK1 pathway was activated (**Figure 8F**). Moreover, cytoskeletal remodeling occurred in the pcDNA3.1-Aldob group (**Figure 8H**), and the podocytes’ motility was higher (**Figure 8I**). Finally, to prove the protective role of FBP in podocytes, we treated podocytes with HG and FBP (5 mM and 10 mM). Western blotting showed that HG induced RhoA/ROCK1 activation was inhibited by FBP (**Figure 8J**) and the cytoskeletal remodeling and motility effect in podocytes under HG conditions were ameliorated (**Figures 8K, L**).

### FBP Administration Protected Diabetic Mice From Foot Process Fusion in Podocytes

**Figure 9:**
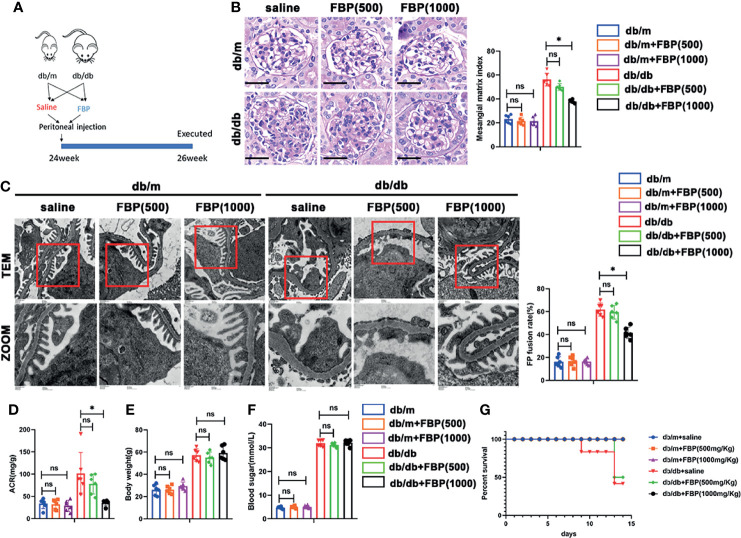
FBP protected db/db mice from foot process fusion in podocytes. **(A)** Schematic diagram of FBP administration in advanced db/db mice. **(B)** Representative microscopy images and quantification of PAS staining of kidney sections from individual group (original magnification ×400). **(C)** Representative transmission electron microscopy images of the ultrastructure of capillary loops in each group (original magnification×8,000, ×12,000). **(D)** Quantitative of ACR (albumin-to-creatinine ratio) in each group. **(E)** Body weight of mice in each group. **(F)** Blood sugar of mice in each group. **(G)** Survival curve of db/m and db/db mice treated with FBP over time. For all figures, n=6 independent experiments/group. *P < 0.05, ns, No significance. Scale bars: 20 µm, FBP, fructose-1,6-bisphosphate; 500, 500mg/kg/d; 1000, 1000mg/kg/d.

To further investigate the role of FBP *in vivo*, we treated db/m and db/db mice with FBP intraperitoneal injection (**Figure 9A**). Surprisingly, we found that in db/db mice treated with a high dose of FBP, glomerular lesions (**Figure 9B**), the fusion of foot processes (**Figure 9C**), urinary ACR (**Figure 9D**) and mortality (**Figure 9G**) were reduced. However, there were no significant changes in blood glucose (**Figure 9F**) and body weight (**Figure 9E**) in mice after FBP treatment. This part of our research indicates that FBP may protect db/db mice from foot process fusion in podocytes and can be potential medications for DKD.

## Discussion

In this study, we observed abnormalities in glycolysis and disorder in the cytoskeletal structure of podocytes under HG stimulation. We identified PFKP as playing a novel endogenous protective role against diabetic injury in podocytes through its catalyzed metabolite FBP. *In vivo* studies showed the activation of PFKP in different models and the detrimental effects of the inhibition of PFKP. Mechanistically, our *in vitro* studies showed that FBP could ameliorate HG-induced cytoskeletal remodeling in podocytes.

In the past few decades, molecular targets for DKD treatment have been intensively investigated (3). However, there are no specific agents for the prevention and treatment of early DKD. Hyperglycemia, which is known to be a crucial part of the pathogenesis of DKD, can damage podocyte function and structure in many ways, thus leading to the occurrence of proteinuria (5, 6), but the underlying mechanisms remain unclarified.

Alteration of glucose metabolism in podocytes under diabetic conditions has received much attention in recent years. Altered glycolytic flux has been postulated to induce a series of transcriptional and translational changes that lead to DKD (23). Moreover, the activation of glycolysis has been proven to have positive effects in DKD patients (8, 10). A study focused on PKM2, which is another key enzyme in the glycolysis process, found that PKM2 activation could protect podocytes from HG-induced injury by protecting mitochondria (10). In our mRNA array, we detected changes of glycolysis enzyme mRNA levels, including the increased level of *Pfkp* and *Hk1*, and decreased in *Pkm* and *Ldha*, indicating the dysfunction of glomeruli glycolysis in db/db mice and the accumulation of glycolysis intermediates. PFK1 is one of the key glycolytic enzymes that catalyzes the conversion of F-1-P to FBP. To date, three subtypes of PFK1 have been identified in vertebrate genomes, termed PFKM (muscle type), PFKL (liver type) and PFKP (platelet type), according to the site of discovery, among which PFKP plays the leading role in the kidney (24). As a major isoform of PFK-1 in cancer glycolysis, PFKP has become an emerging anticancer target (25). Here we found that PFKP showed the highest expression level and was significantly upregulated in the glomeruli of db/db mice. Consistently, PFKP was upregulated in DKD patients compared with control renal tissues obtained from cancer patients, the result was surprising because PFKP was previously reported to be upregulated in cancer (25). In addition, we detected increased ACR level, mortality, and more severe foot process fusion in db/db mice when treated with CTZ, which is an inhibitor of PFKP (21). Besides, the use of FBP, a catalysate of PFKP, improved proteinuria and renal injury in db/db mice. These results indicate that PFKP may play the role of endogenous protective factors.

In the last few decades, studies have shown that the actin cytoskeleton directly binds to many metabolic enzymes, including PFK-1, ALDO and glycerol triphosphate dehydrogenase (GAPDH), and recent studies have shown that remodeling and assembly of the actin cytoskeleton is a major energy-consuming process, accounting for approximately 50% of ATP consumption (12). It was reported that on the soft extracellular matrix, the active E3 ubiquitin ligase TRIM21 binds and ubiquitinates PFK-1 in the cytoplasm, resulting in targeted degradation of PFK-1 and thereby maintaining glycolysis at a relatively low level. When cells move to the hard extracellular matrix, the contraction force is increased, F-actin and Talin binding of bound integrins is also increased, thereby stimulating actin-binding and TRIM21 chelation. Inactivation of this process results in PFK-1 accumulation in the cytoplasm and increases glycolysis (13). Furthermore, FBP is a high-energy glycolytic intermediate that exerts protective action against many harmful conditions in various cell types and tissues through its anti-inflammatory, immunomodulatory and neuroprotective properties (26). Moreover, it can regulate the activation of Ras, which is a major regulator of cell proliferation (27). Additionally, FBP mediates glucose sensing by AMPK (28), and AMPK signaling can increase the phosphorylation of vinculin at Y822, which triggers the activation of the RhoA–ROCK–MLCK–MLC pathway, culminating in the reinforcement of the actin cytoskeleton (12). AMPK is also the energy sensor in cells and is activated when cells are starved of nutrients. Local activation of AMPK increased ATP production, mitochondrial flux, and cytoskeletal dynamics, and excessive energy may promote actin cytoskeletal remodeling (29). In addition, FBP can promote a feedback loop between PFK1, phosphatidylinositol-3-kinase/protein kinase B (PI3K/Akt), and PFK2/PFKFB3 in T cells (30), and the PI3K/Akt signaling pathway is closely related to the podocyte cytoskeleton (31). Hence, FBP is involved in the regulation of cytoskeletal structure.

Cytoskeletal architecture in podocytes is pivotal to its function (5, 11). Cytoskeletal remodeling in podocytes contributes to the progression of DKD (32, 33), as the assembly of F-actin is associated with the formation and migration of podocyte foot processes. Treatment targeted toward the cytoskeletal architecture has been proven effective in glomerular diseases (34). Small GTPases that belong to the Ras homology (Rho) family play essential roles in regulating cell migration by controlling cytoskeletal system (35). RhoA is the most important member of the Rho family, and its major downstream effector is Rho-associated kinase (ROCK), which is a serine/threonine kinase, is highly involved in the biological processes of cell movement, cell migration, gene transcription, nerve regeneration, and apoptosis (36). ROCK1 is localized to the plasma membrane and plays an important role in cell movement, and RhoA is critical for the recruitment of ROCK1 to the plasma membrane (37). It has also been found that RhoA/ROCK1 pathway contributes to the development of DKD (22). Moreover, inhibition of RhoA/ROCK1 pathway prevents the occurrence of pathologic changes in DKD *in vivo* (38). We found that the suppression of PFKP with siRNA resulted in lower FBP levels and more severe cytoskeletal remodeling in podocytes *in vitro*. Conversely, overexpression of PFKP in the HG environment relieved cytoskeletal remodeling and increased FBP production. Furthermore, inhibition of ALDOB can ameliorate podocyte damage by elevating FBP levels. In contrast, the activation of ALDOB reduced FBP production and led to increased cytoskeletal remodeling. More importantly, FBP addition rescued podocytes from cytoskeletal remodeling under HG conditions and could alleviate the renal injury of db/db mice *in vivo*.

To our knowledge, the present study demonstrates for the first time that glucose metabolism is associated with the cytoskeletal remodeling in podocytes through ROCK1/RhoA signaling pathway. Suppression of PFKP in podocytes exacerbates diabetic kidney injury and cytoskeletal remodeling in podocytes. PFKP, the key regulator of glucose metabolism, affected cytoskeletal structure by regulating FBP levels. These findings provide evidence that PFKP may be a potential target for podocyte injury in DKD and provide a rationale for applying glycolysis enhancing agents in patients with DKD.

### Limitations of Study

The protective role of PFKP against DKD is revealed here only in male mice. Whether PFKP has the same function in female mice has not been investigated. We did not use *Pfkp* gene overexpression or knockout mice to perform our experiments. While we demonstrate that FBP directly affects the RhoA/ROCK1 pathway, how FBP influences it deserves to be further investigated. Lastly, whether PFKP and FBP protect against DKD in clinical situations, or at least in nonhuman primates, remains to be further investigated.

## Data Availability Statement

The datasets presented in this study can be found in online repositories. The names of the repository/repositories and accession number(s) can be found below: Gene Expression Omnibus GSE184836, https://www.ncbi.nlm.nih.gov/geo/query/acc.cgi?acc=GSE184836.

## Ethics Statement

The studies involving human participants were reviewed and approved by the Research Ethics Committee of Renmin Hospital of Wuhan University. The patients/participants provided their written informed consent to participate in this study.

## Author Contributions

ZWZ, WL, and GD participated in research design. ZWZ, QL, HH, KY, ZJZ, and JH performed experiments. ZWZ, QL, ZC, JZ, JF, QC, and WL performed data analysis and interpretation; ZWZ and WL drafted the paper. ZWZ, JH, and GD designed and supervised the studies. All authors contributed to the article and approved the submitted version.

## Funding

This work was supported by grants from the National Natural Science Foundation of China (81970631 to WL and 82070713 to GD).

## Conflict of Interest

The authors declare that the research was conducted in the absence of any commercial or financial relationships that could be construed as a potential conflict of interest.

## Publisher’s Note

All claims expressed in this article are solely those of the authors and do not necessarily represent those of their affiliated organizations, or those of the publisher, the editors and the reviewers. Any product that may be evaluated in this article, or claim that may be made by its manufacturer, is not guaranteed or endorsed by the publisher.
