# Supplementary material for: PFKP Activation Ameliorates Foot Process Fusion in Podocytes in Diabetic Kidney Disease
Source: Front Endocrinol (Lausanne). 2022 Jan 14;12:797025. doi: 10.3389/fendo.2021.797025 (PMC8794994; doi:10.3389/fendo.2021.797025)

**Supplementary**

**Figure S1 Blood sugar changes in db/m and db/db mice treated with CTZ**

**
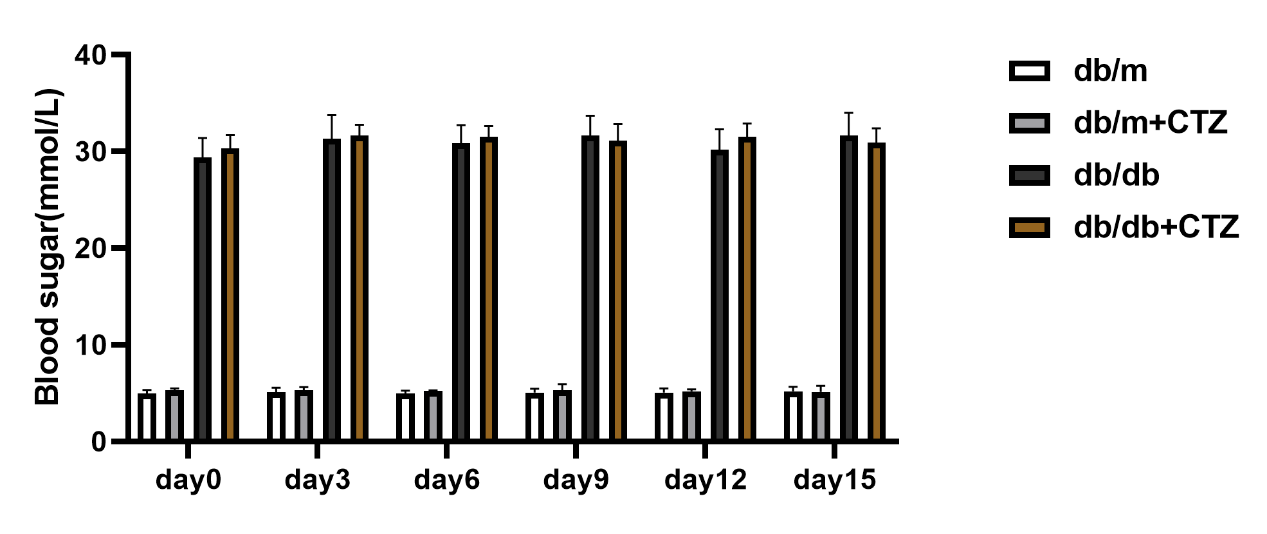
**

**Figure S2. Body weight changes in db/m and db/db mice treated with CTZ
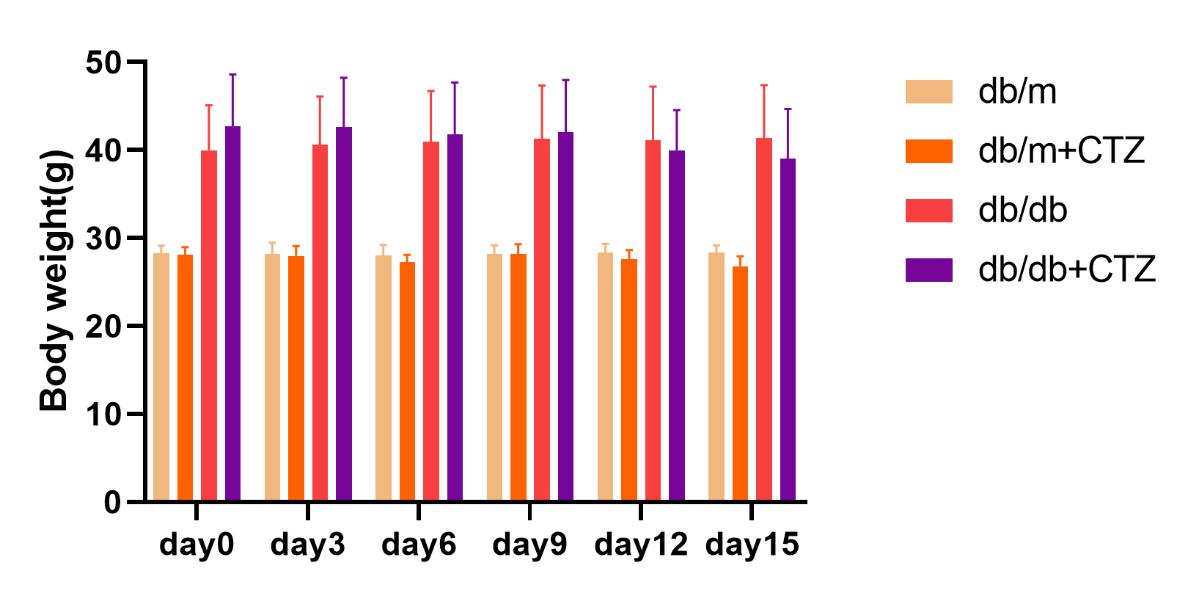
**

**Figure S3. PFK expressions in podocytes**


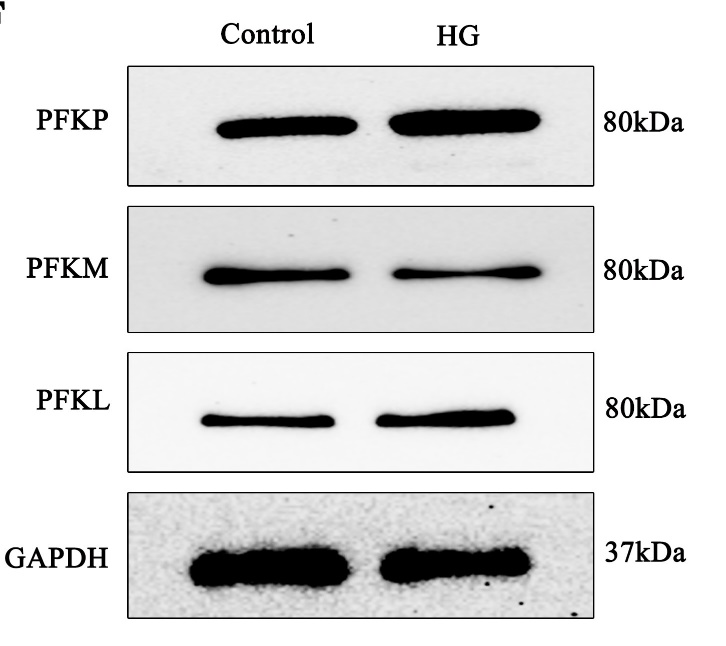


**Figure S4. PFK mRNA expressions in podocytes**


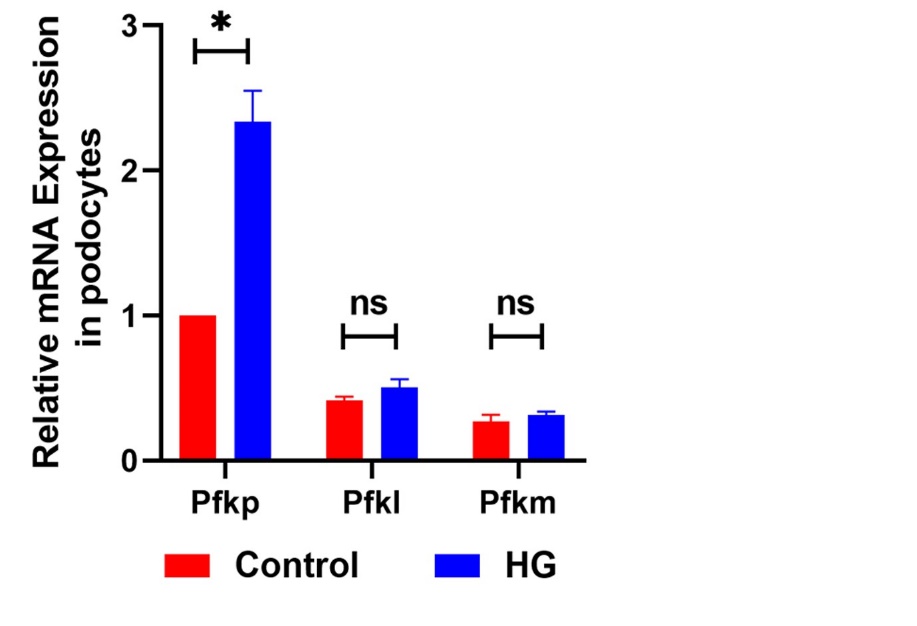


**Figure S5. ALDO mRNA expressions in podocytes**


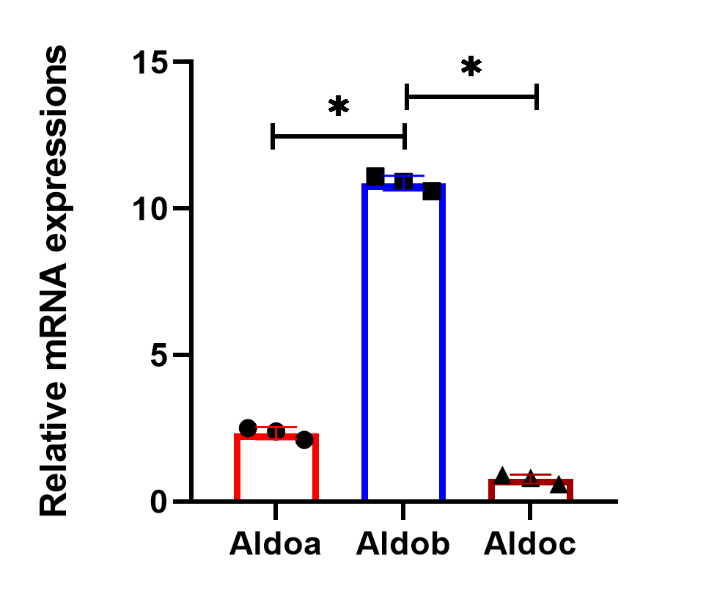

Supplement: Supplementary file 1 [file DataSheet_1.docx]
