# Supplementary material for: PFKP Activation Ameliorates Foot Process Fusion in Podocytes in Diabetic Kidney Disease
Source: Front Endocrinol (Lausanne). 2022 Jan 14;12:797025. doi: 10.3389/fendo.2021.797025 (PMC8794994; doi:10.3389/fendo.2021.797025)
Supplement: Supplementary file 3 [file Table_1.docx]

**Table S1.** Clinical information of the patients

| number | age(year) | gender | group | acr(mg/g) | UTP(g/24h) | SCr(umol/L) |
| --- | --- | --- | --- | --- | --- | --- |
| 1 | 47 | female | control | 23 | 0.05 | 67 |
| 2 | 50 | female | control | 18 | 0.12 | 76 |
| 3 | 48 | female | control | 16 | 0.07 | 57 |
| 4 | 55 | male | control | 21 | 0.08 | 54 |
| 5 | 45 | male | control | 12 | 0.03 | 56 |
| 6 | 50 | male | control | 17 | 0.11 | 70 |
| 7 | 45 | female | DKD | 3093 | 1.2 | 123 |
| 8 | 37 | female | DKD | 4015 | 1.5 | 156 |
| 9 | 42 | female | DKD | 4291 | 1.8 | 200 |
| 10 | 56 | male | DKD | 5678 | 2.15 | 201 |
| 11 | 48 | male | DKD | 5443 | 3.2 | 145 |
| 12 | 49 | male | DKD | 7834 | 5.8 | 189 |

**Table S2.** Primers used in Real-time PCR

| Gene | Species | Forward | Reverse |
| --- | --- | --- | --- |
| *Pfkp* | human | AGGCAGTCATCGCCTTGCTAGA | ATCGCCTTCTGCACATCCTGAG |
| *Pfkl* | human | AAGAAGTAGGCTGGCACGACGT | GCGGATGTTCTCCACAATGGAC |
| *Pfkm* | human | CTGTTCGCTCTACCGTGAGGAT | TTGGAACCACCTTGACCAGTCC |
| *Gapdh* | human | GTCTCCTCTGACTTCAACAGCG | ACCACCCTGTTGCTGTAGCCAA |
| *Aldob* | human | AGCCCTCGCTATCCAGGAAAACG | TGGCAGTGTTCCAGGTCATGGT |
| *Aldoc* | human | CATTCTGGCTGCGGATGAGTCT | CACACGGTCATCAGCACTGAAC |
| *Aldoa* | human | GACACTCTACCAGAAGGCGGAT | GGTGGTAGTCTCGCCATTTGTC |
| *Pfkp* | mouse | AAGAGGAAACCAAGCAGTGCGC | TTCCTCGGAGTTTCACGGCTTC |
| *Pfkl* | mouse | CCATCAGCAACAATGTGCCTGG | TGAGGCTGACTGCTTGATGCGA |
| *Pfkm* | mouse | GCTTCTAGCTCATGTCAGACCC | CCAATCCTCACAGTGGAGCGAA |
| *Ldha* | mouse | ACGCAGACAAGGAGCAGTGGAA | ATGCTCTCAGCCAAGTCTGCCA |
| *Pkm* | mouse | CAGAGAAGGTCTTCCTGGCTCA | GCCACATCACTGCCTTCAGCAC |
| *Hk1* | mouse | GAAAGGAGACCAACAGCAGAGC | TTCGTTCCTCCGAGATCCAAGG |
| *Gapdh* | mouse | CATCACTGCCACCCAGAAGACTG | ATGCCAGTGAGCTTCCCGTTCAG |
